# High Density Nodes in the Chaotic Region of 1D Discrete Maps

**DOI:** 10.3390/e20010024

**Published:** 2018-01-04

**Authors:** George Livadiotis

**Affiliations:** Space Science & Engineering, Southwest Research Institute, San Antonio, TX 78238, USA; glivadiotis@swri.edu; Tel.: +1-210-274-4028

**Keywords:** bifurcation, chaos, critical lines, logistic map, nodes, Feigenbaum constant, entropy

## Abstract

We report on the definition and characteristics of nodes in the chaotic region of bifurcation diagrams in the case of 1D mono-parametrical and S-unimodal maps, using as guiding example the logistic map. We examine the arrangement of critical curves, the identification and arrangement of nodes, and the connection between the periodic windows and nodes in the chaotic zone. We finally present several characteristic features of nodes, which involve their convergence and entropy.

## 1. Introduction

Let the one-dimensional, mono-parametrical and S-unimodal map (difference equation), $x_{t+1}=f\left(x_{t};p\right)$, where $x_{t}=f^{\left(t\right)}\left(x_{0};p\right)$ is the *t*th iterated map, *x*_0_ is the initial value, and *p* is the nonlinear parameter. As an example, we consider the Logistic map [1,2,3,4,5,6,7,8,9,10,11,12,13,14,15,16]:

(1) $$x_{t+1}=f(x_{t};p),\;\mathrm{with}\;f\left(x_{t};p\right)=p\cdot x_{t}\cdot \left(1-x_{t}\right)$$

A bifurcation diagram is separated into two parts, the zone of Order, *p* < *p*_∞_, [3,4,5,17,18,19,20,21,22], where only periodic orbits may occur, and the zone of Chaos, *p* > *p*_∞_, [3,6,7,13,17,18,23,24,25,26,27,28,29,30], where the chaos appears, and both chaotic and periodic orbits may occur; *p*_∞_ is the Feigenbaum’s point [4,9,31], which defines the boundary point of the two zones.

**Figure 1:**
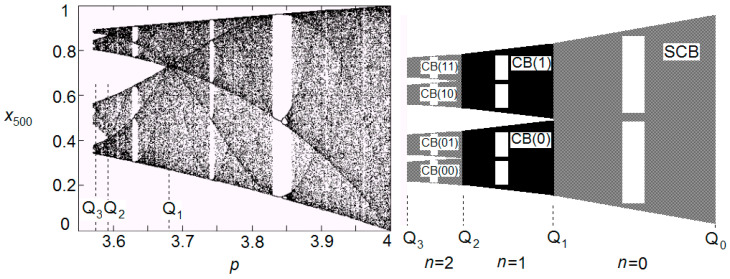
Division or merge of CB sections: (**Left**) The chaotic zone of the Logistic bifurcation diagram after *t* = 500 iterations. (**Right**) Sketch of the chaotic zone, indicating the numbering of CBs (the window of period three is also sketched; of course, the shapes and scales of the figure are not realistic). In both diagrams, we indicate the CB’s generation *n* and band-mergings {*Q_n_*}. (Note: There is an infinite number of WOMs in each CB, but we only sketch the WOM of period 3 for simplicity. For the same reason, the three bifurcation miniature diagrams, located within the WOM of period 3, are illustrated with a simple straight vertical line.) (Taken from [9]).

The Chaotic zone of a bifurcation diagram can be further separated in the sections named as Chaotic Bands (CB) (Figure 1). As the nonlinear parameter *p* decreases, it reaches a boundary point, where each section is divided into two new CBs [9,32,33]. In the sketched part on the right of Figure 1, at the boundary point *p* = *Q*_2_, the CB(0) is divided into two new CBs, CB(00) and CB(01), while the CB(1) is divided into CB(10) and CB(11). The binary system is applied in the numbering, i.e., CB(0), CB(1), CB(00), CB(01), CB(10), CB(11), and so on. The Single Chaotic Band (SCB) is the initial section prior to any splitting [13,15,19,31,32,33,34,35,36,37,38]. This phenomenon of CB division is better known as “merging”. Indeed, while the division of CBs appears as *p* decreases, a merge of CBs is observed as *p* increases. The value of *p*, for which the division or merge of a CB takes place, is symbolized as *Q_n_* and is called band-merging (Figure 1) [9,13,15,22,32,39,40,41,42,43,44,45,46,47,48,49]; *n* is the generation of each band-merging as *p* decreases. In the chaotic zone, we have the reverse bifurcation, that is, a bifurcation of the CBs as *p* decreases [13,15,47,50]. The self-similarity of this reverse cascade was studied using the Lyapunov Characteristic Number (*LCN*) [13,15,22,33,47,51,52,53].

**Figure 2:**
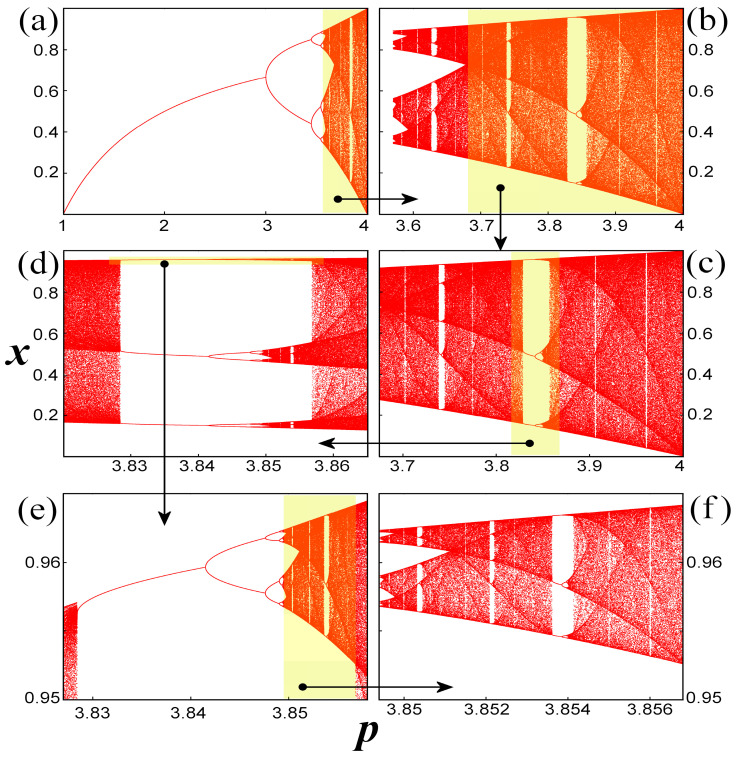
(**a**) Main bifurcation diagram for 1 ≤ *p* ≤ 4 (main zones of order and chaos). (**b**) Main chaotic zone. (**c**) Single Chaotic Band (SCB), that is, the basic unit being repeated in smaller scales in the reverse period-doubling cascade. (**d**) WOM of period 3 in SCB. (**e**) Upper periodic attractor inside the WOM of period 3 and the produced secondary bifurcation diagram, a miniature of the main bifurcation diagram. (**f**) Secondary chaotic zone of the upper periodic attractor inside the WOM of period 3. The similarities between the main chaotic zone in (**b**) and the miniature chaotic zone in (**e**) are remarkable. The arrangements of WOMs, critical curves, and nodes, are some of the common features of the main and miniature chaotic zones. (Notes: Each of the colored indicated areas is magnified in the respective sequential panel. The diagrams are computed for 10^6^ iterations.). (Taken from [16]).

Within the chaotic zone and along the axes of the nonlinear parameter, *p*, chaos interchanges with islands of stability, which appear as Windows of Organized Motion (WOM) or periodic windows within chaos [1,9,13,14,15,16,19,22,32,34,35,36,39,42,54,55,56,57,58,59,60,61]. Each WOM is characterized by periodic orbits, with the least one defining the characteristic period of each WOM: A WOM of period *T* starts with a number of *T* fold bifurcations leading to *T* pairs of orbits (1 stable and 1 unstable). The topology of periodic orbits inside each WOM forms a number of complete bifurcation diagrams in miniature that equals the WOM period (Figure 2). Therefore, aside from the main zones of order and chaos, which have already been referred, secondary zones of order and chaos exist also for *p* > *p*_∞_, inside each WOM [9,51]. The closing of a window takes place when the orbits can escape from the regions of the miniature chaotic zones, merging into the main chaotic zone [61].

A certain chaotic orbit inside WOM of period 3 (or, briefly, WOM-3) in SCB has total period equal to 1 × 3 = 3, where 1 stands for the iterations needed for the orbit to visit SCB, that is trivially, one iteration. However, in the case of higher generation CBs, e.g., CB(0) or CB(1), the orbits visit a certain CB every second iteration. Namely, chaotic orbits visit WOM-3 in CB(0) or CB(1) every second iteration. Hence the period of the orbit is double the period of the WOM, i.e., in the examined case the total period is 2 × 3. In general, the period of the orbit in a WOM of period *T* in a CB of generation *n* is *n* × *T*. (See also [13,16]).

Unimodal maps which are defined on the unit interval, have one differentiable maximum, and fall off monotonically on both sides, have a common property called *structural universality* [24,26,27,47]. The maps with this universality have their infiniteness of WOMs to appear with the same arrangement, in any CB of the Chaotic zone. This property let to the classification of WOMs using symbolic dynamics [24,26,27,32,39,47,59,62,63].

The purpose of this paper is to study the arrangement and features of nodes, high density points in the chaotic zone, which are interwoven with the location and period of WOMs. In our analysis, we focus on SCB, but similar features characterize all CBs. In Section 2, we examine the critical curves that appear in the chaotic zone, using the density of the variable *x*. In Section 3, we examine the identification and arrangement of nodes in the chaotic zone, separating those in two types: primary and secondary ones; each type has different arrangement and features. In Section 4, we derive the mathematical forms of critical curves and primary nodes. In Section 5, we show the connection of nodes with the universal arrangement of WOMs, while, in Section 6, we compute several characteristic features of nodes. Finally, Section 7 summarizes the conclusions, while, in Appendix A, are all tables mentioned in the main text.

## 2. Critical Lines in the Chaotic Zone

**Figure 3:**
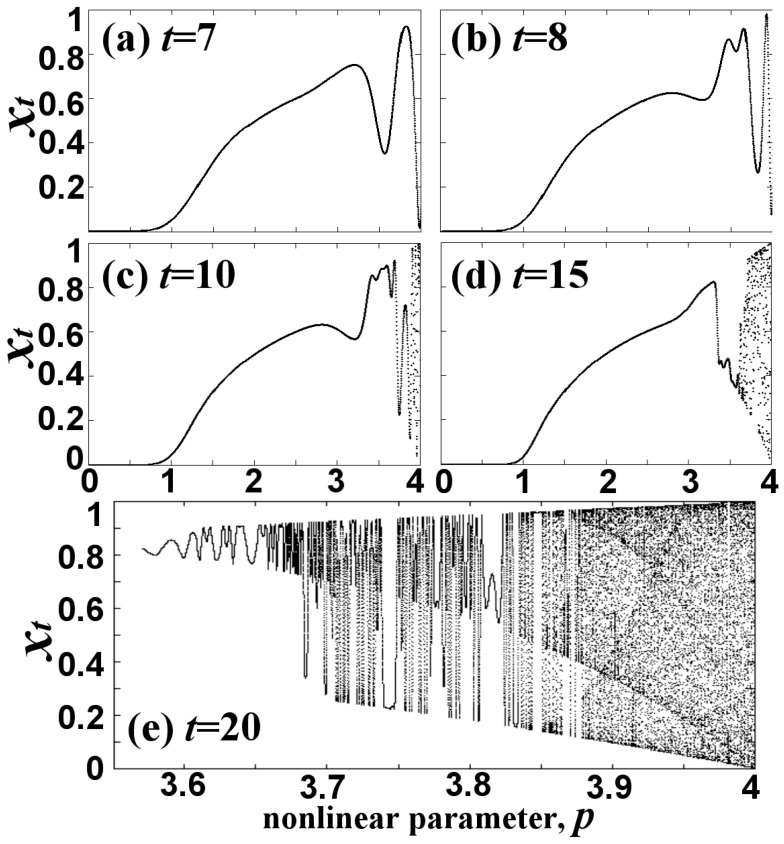
Formation of the chaotic zone as the number of iterations increases.

As we observe in Figure 1a, the chaotic zone is characterized by Critical Curves (CC), which are the loci of enhanced density of points. Figure 3a–e shows the formation of the chaotic zone as the number of iterations increases. We observe the multi-folding of chaotic orbits in the chaotic zone, while the critical curves appear to be the locus of the extrema of the orbits due to their folding, as shown in Figure 3e. This is reasonable because the density of mapped points P(*x*; *p*) is, by construction, inversely proportional to the derivative of the map $\partial x_{t}/\partial p=\partial f^{\left(t\right)}\left(x_{0};p\right)/\partial p$.

The critical curves can be expressed by

(2a) $$X(p;n)=f^{\left(n+1\right)}\left(\frac{1}{2};p\right)$$

(2b) $$i.e.,\;X(p;0)=\frac{1}{4}p,\;X(p;1)=\frac{1}{16}p^{2}(4-p),\;X(p;2)=\frac{1}{256}p^{3}(4-p)(p^{3}-4p^{2}+16),\;\mathrm{etc}.$$

where *n* is the multiplicity of the critical curves. (The derivation of Equation (2a) is shown in Section 4).

The critical curves of multiplicity *n* = 0 and *n* = 1 correspond to those surrounding the chaotic zone at its upper and lower edge, respectively. The curve of multiplicity *n* = 2 is the dominant one located in the interior of SCB, passing through the band-merging *Q*_1_ and the end of the chaotic zone at the point (*p* = 4, *x* = 0). (In fact, all critical curves pass through the point (*p* = 4, *x* = 0), except the chaotic orbit of *n* = 0, which passes through the point (*p* = 4, *x* = 1)).

**Figure 4:**
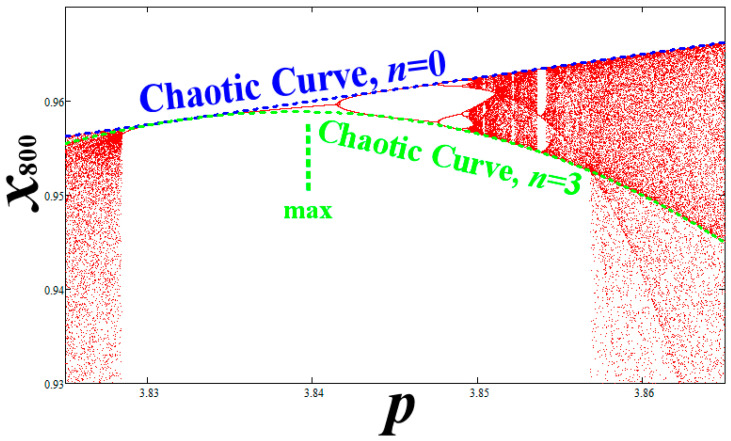
Similar to Figure 3e, we plot the upper periodic attractor inside WOM of period 3 (and the produced secondary bifurcation diagram), and co-plot the critical curves with multiplicity *n* = 0 (blue) and *n* = 3 (green). We observe that the chaotic curves of the main chaotic zone appear also in miniature chaotic zones inside WOMs but with smaller multiplicity. Indeed, the critical curve with multiplicity *n* = 3 appears to pass through the lowest *x*-values of the miniature chaotic zone, that is, the role of the critical curve with multiplicity *n* = 1. What is happening is that if *n* is the multiplicity of a critical curve in the main chaotic zone, then in a WOM of period *T*, the same critical curve becomes of multiplicity *n*/*T*. If this ratio is less than 1, then the critical curve does not appear in that WOM.

All the higher ranking critical curves (*n* ≥ 3) have maxima and minima located inside WOMs of periods equal to the multiplicity of the curves. It must be noted that these maxima or minima of the critical curves are located inside the miniature’s zones of order (not exactly at the branches). However, the critical curves of the chaotic main bifurcation diagrams have similar role in the miniature chaotic zones, but with different multiplicity (Figure 4). For example, the curve of *n* = 3 has a single maximum; this is located inside the WOM of period 3. This WOM starts at *p* = 2√2 + 1 = 3.82842712474619, the double period bifurcation starts at *p* ≈ 3.8414 …, while the maximum is located at *p* ≈ 3.8390 …. The chaotic curve of multiplicity *n* = 3 in the main chaotic zone becomes of multiplicity *n* = 3:3 = 1 in the secondary chaotic zone (that is the critical curve passing through the lowest *x*-values). It is interesting that, in general, the product of the multiplicity of a critical curve with the period of the WOM is a constant. In the example of WOM-3, multiplicity is 3 when periodicity is 1 (main chaotic zone), while multiplicity is 1 when periodicity is 3, i.e., 3 × 1 = 1 × 3. Consequently, the critical curves, with multiplicity *n* in the main chaotic zone, do not appear in WOMs with period larger than *n*.

Similarly, the critical curve of *n* = 4 has a single maximum located at the WOM of period 4, at *p* = 3.960101882689952. However, the curve of *n* = 5 has three maxima, each located to one of the three WOM of period 5 that exist in SCB. The critical curve of *n* = 6 also has three maxima at SCB, corresponding to the three WOMs of period 6 in SCB. However, there is one more maximum, which is located at the WOM of period 3 in the chaotic bands of *n* = 1, i.e., CB(0) and CB(1), where the chaotic orbits have period doubled than the respective ones in SCB, i.e., the chaotic orbits at the WOM of period 3 correspond to a total period 2 × 3.

**Figure 5:**
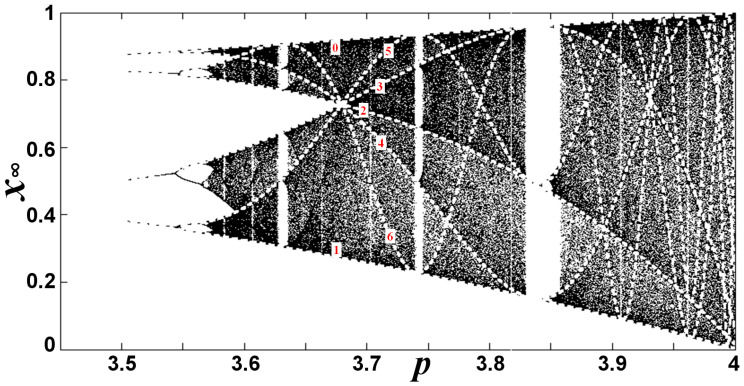
Plot of the chaotic zone and critical curves of multiplicity 0–6, as modelled in Equation (2).

Figure 5 plots the chaotic zone, together with the critical curves modelled by Equation (2). We observe the curves of multiplicity from 0 to 6 in SCB. We also observe some other characteristic property of these curves, that is, their divergence from and convergence to points called nodes.

## 3. Identification and Arrangement of Nodes in the Chaotic Zone

The density of the map values, *x_t_*, after *t* iterations of an initial value *x*_0_ in the interval (0, 1), denoted by P(*x*; *p*), is given by (e.g., see: [10,22,33]):

(3) $$P\left(x;p\right)\equiv \lim_{\begin{array}{l} N\to \infty \\ dx\to 0 \end{array}}\frac{dN\left(x-dx/2\leq x_{t}\leq x+dx/2\right)}{N\cdot dx}$$

**Figure 6:**
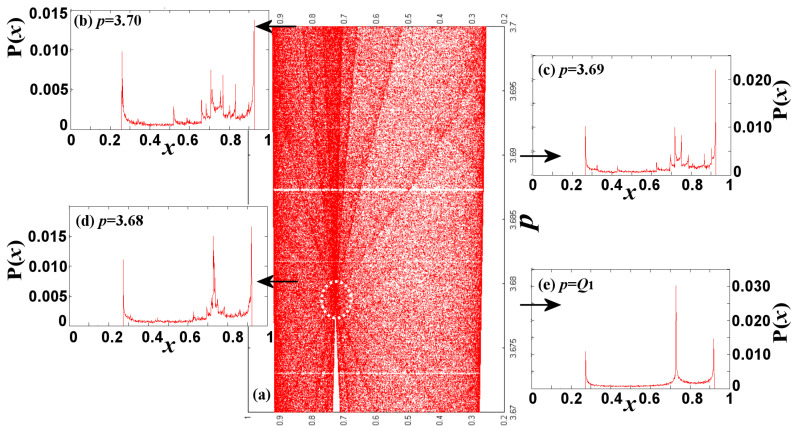
Density profiles near the node of order 0 (band-merging *Q*_1_), for: (**a**) *p* = 3.7; (**b**) *p* = 3.69; (**c**) *p* = 3.68; and (**d**) *p* = *Q*_1_. (**e**) The chaotic band SCB near the node of order 0, *N*_0_.

Next, we construct the density P(*x*; *p*) for nonlinear parameter values taken at the beginning of SCB. In Figure 6b–e, we start with *p* = 3.7, and then, decrease *p* until we reach *Q*_1_, located at *p* = 3.678573510428320. Each density peak indicates a critical curve, cut at the certain value of *p*. We observe that there are plenty of peaks, most of them undistinguished from each other, but as the nonlinear parameter approaches *Q*_1_, they are all accumulated into three main peaks; two of these correspond to the peaks at the upper and lower edges of SCB, while the third corresponds to a node, located at the point that connects CB(0), CB(1), and SCB. This node is shown in Figure 6a that magnifies the local region of chaotic zone. We observe that the node is a point of intersection of critical curves.

The node, identified in Figure 6e, corresponds to a density profile at *p* = *Q*_1_ with exactly three peaks, two at the upper/lower edges, and one at the interior. For some larger value of the nonlinear parameter, *p* = 3.9277337001786751, there is a node corresponding to a density profile with four single peaks. As we will see further below, CBs have nodes that correspond to density profiles with any number of peaks; the nodes, the number of the peaks at their density profiles, and their arrangement in the CBs of the chaotic zone, are interwoven with the period and arrangement of WOMs.

**Figure 7:**
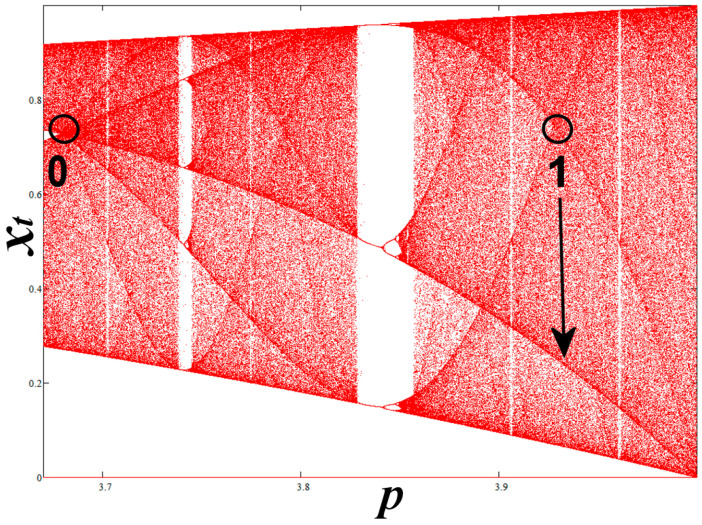
The single chaotic band (SCB), where we observe the encircled primary nodes of order 0 and 1. The arrow indicates the critical curve responsible for the nodal order 1.

We rank the nodes as follows: The order of a node is given by the number of the peaks that surround the node in the corresponding density profile in the interior of the CB, namely, excluding the two edges and the node itself. Therefore, the node at *p* = *Q*_1_ ≡ *N*_0_ is of order 0, while the node at *p* = 3.9277337001786751 ≡ *N*_1_ is of order 1. Both nodes are shown in the SCB plotted in Figure 7.

**Figure 8:**
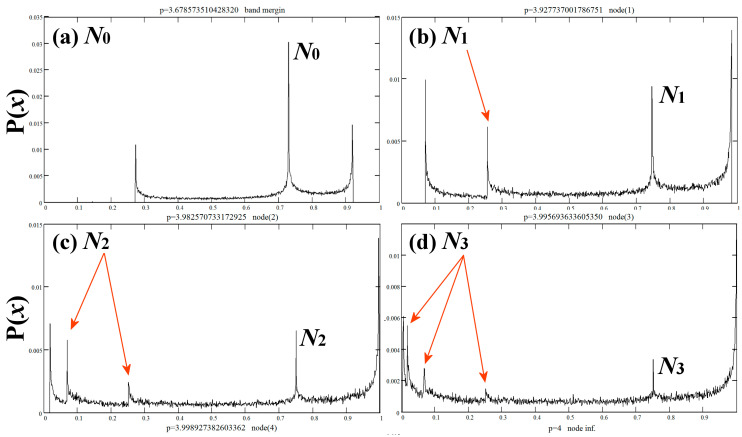

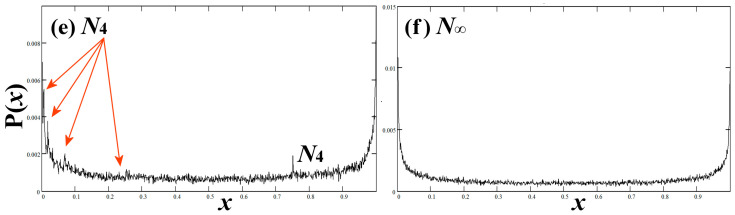
Density profiles at primary nodes of order 0–4 and ∞. The arrows indicate the density peaks that correspond to critical curves (and not to the edges or the node itself), whose number defines the nodal order.

As the nonlinear parameter *p* increases beyond the node of order 1, we find a node of order 2 at *p* = 3.982570733172925 ≡ *N*_2_. In general, there is only one node of order *n* + 1 at the right-hand side of the node of order *n*. Thus, the location of these nodes at the nonlinear parameter axis is arranged as follows: *N*_0_ < *N*_1_ < *N*_2_ < … < *N_n_* < *N_n_*_+1_ < … < *N_∞_* = 4. These types of nodes are called primary. Table A1 in Appendix A contains the positions *N_n_* of primary nodes, up to the order 8. Figure 8 plots the density profiles at the band-merging, the primary nodes up to the 4th order, and the case of fully developed chaos at *p* = 4, which corresponds to the limit where all the primary nodes converge and is symbolized as *N_∞_*.

## 4. Mathematical Formulae of Critical Curves and Nodes

We construct the mathematical formulae that give the critical curves and the position of each primary node. We start by partitioning the map in a sequence of subintervals, ${\left\{I_{n}\right\}}_{n=0}^{\infty }$, where *I_n_* involves orbits with a specific number of *n* successive ascents before a descent occurs [16].

**Figure 9:**
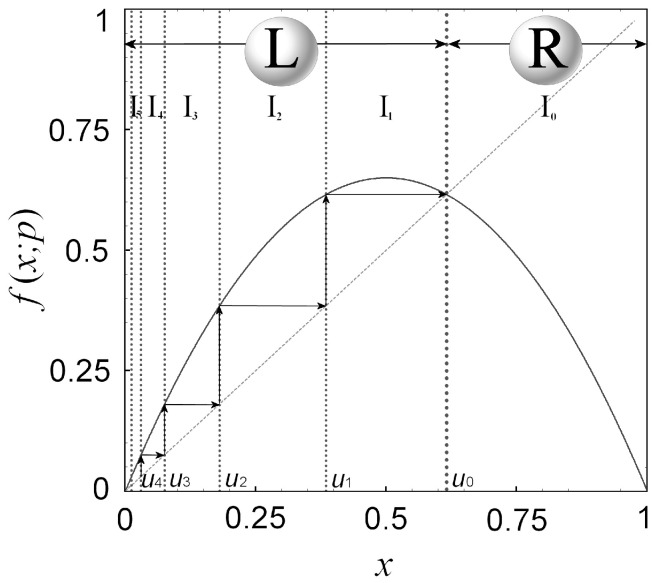
Sequence of the subintervals ${\left\{I_{n}\right\}}_{n=0}^{\infty }$ and their boundary points ${\left\{u_{n}\right\}}_{n=0}^{\infty }$. (Taken and modified from [16]).

The fixed point given by $f\left(u_{0}\right)=u_{0}$ separates the whole interval 0≤x≤1 in left-hand (L) and right-hand (R) sides, i.e., $0\leq x<u_{0}$ and $u_{0}<x\leq 1$, respectively (Figure 9). Let $u_{n+1}$ be the preimage of $u_{n}$, ∀n≥0, defined by the reverse map, $u_{n+1}={f^{-1}}_{L}(u_{n})$, where ${f^{-1}}_{L}(u_{n})$ is the one of the two single-valued inverse functions of f(x): The inverse, $f^{-1}(x)$, is a bi-valued function in the map domain [0, 1], while the single-valued functions, ${f^{-1}}_{L}(u_{n})$ and ${f^{-1}}_{R}(x)$, defined in $0\leq x<u_{0}$ and $u_{0}<x\leq 1$, respectively. Thus, the preimage points constitute a sequence ${\left\{u_{n}\right\}}_{n=0}^{\infty }$, with $u_{n}={{f^{-1}}_{L}}^{\left(n\right)}(u_{0})$, with an accumulation limit point at $u_{\infty }=\underset{n\to \infty }{\lim}{{f^{-1}}_{L}}^{\left(n\right)}(u_{0})=0$.

If $x_{n}=f^{\left(n\right)}(x_{0})$ is in (R), the orbit lies always below the diagonal, hence, only one descent is conceivable before the next sequence of ascents occurs, i.e., $x_{n+1}=f^{\left(n+1\right)}(x_{0})<x_{n}=f^{\left(n\right)}(x_{0})$. (Note: An ascent or a descent of an orbit is its jump to higher or lower *x*-values after one iteration.) If $x_{n}=f^{\left(n\right)}(x_{0})$ is in (L), the orbit lies always above the diagonal, hence, only subsequent ascents can occur. For *n* successive ascents, we have $x<f\left(x\right)<f^{2}\left(x\right)<\cdots <f^{n}\left(x\right)$. All the points of an orbit in subinterval $I_{1}=\left\{x\in \left(u_{1},u_{0}\right)\right\}$ are mapped after one ascent to subinterval $I_{0}=\left\{x\in \left(u_{0},1\right)\right\}$ (that is, the whole interval (R)), and then, from $I_{0}$, they are mapped after a descent back to (L). In addition, all the points of an orbit in subinterval $I_{2}=\left\{x\in \left(u_{2},u_{1}\right)\right\}$ are mapped after one ascent to subinterval $I_{1}=\left\{x\in \left(u_{1},u_{0}\right)\right\}$. In general, all the points of an orbit in subinterval $I_{n}=\left\{x\in \left(u_{n},u_{n-1}\right)\right\}$ are mapped after one ascent to subinterval $I_{n-1}=\left\{x\in \left(u_{n-1},u_{n-2}\right)\right\}$, and so on, until $I_{0}$ is reached (Figure 9).

In this way, the map domain, 0≤x≤1, can be partitioned into subintervals $I_{n}$, where *n* indicates the number of the successive ascents, before a descent occurs, namely:

(4) $$x\in I_{n}\Leftrightarrow x<f(x)<f^{\left(2\right)}(x)<\ldots <f^{\left(n\right)}(x),\;f^{\left(n+1\right)}(x)<f^{\left(n\right)}(x)$$

so that

(5a) $$f(u_{1})=u_{0},\;f^{\left(2\right)}(u_{2})=f(u_{1})=u_{0},\;f^{\left(n\right)}(u_{n})=\cdots =f^{\left(2\right)}(u_{2})=f(u_{1})=u_{0}$$

or

(5b) $$u_{1}={f^{-1}}_{L}(u_{0}),\;u_{2}={f^{-1}}_{L}(u_{1})={{f^{-1}}_{L}}^{\left(2\right)}(u_{0}),\ldots ,u_{n}={f^{-1}}_{L}(u_{n-1})=\ldots ={{f^{-1}}_{L}}^{\left(n\right)}(u_{0})$$

In the case of the logistic map, we find

(5c) $$u_{n+1}={f^{-1}}_{L}(u_{n}),\;{f^{-1}}_{L}(x)=\frac{1}{2}\cdot \left[1-\sqrt{1-(4/p)\cdot x}\right]$$

that is, for up to *n* = 3:

(5d) $$u_{0}=1-1/p,\;u_{1}=1/p,\;u_{2}=\frac{1}{2}\cdot (1-\sqrt{1-4/p^{2}}),\;u_{3}=\frac{1}{2}\cdot \left[1-\sqrt{1-(2/p)\cdot (1-\sqrt{1-4/p^{2}})}\right]$$

The highest *x*-value of the orbit lies in subinterval *I*_0_ for all the values of *p* in SCB (i.e., *Q*_1_ < *p* < *Q*_0_ = 4); at *p* = *Q*_1_, the highest *x*-value is *x*_max_ = *Q*_1_/4, while at *p* = *Q*_0_ = 4, the highest *x*-value becomes *x*_max_ = 1. The maximum of the map is located at (*x* = 1/2, *f*(*x*) = *p*/4), which lies in subinterval *I*_1_; thus, after one ascent, the maximum will be mapped to *I*_0_, reaching the highest possible *x*-value, given by

(6a) $$x_{\max}(p)=f(\frac{1}{2};p)=\frac{1}{4}p$$

Now, the lowest possible *x*-value is reached after a descent of the highest *x*-value, that is, mapped to

(6b) $$x_{\min}(p)=f^{\left(2\right)}(\frac{1}{2};p)=\frac{1}{16}p^{2}(4-p)$$

Further iterations produce the critical curves of higher multiplicity (given in Equation (2)).

It is important to note that not all subintervals *I_n_* exist for a certain value of the nonlinear parameter *p* in SCB. As *p* increases, chaotic orbits reach higher and lower *x*-values. While the highest point will be always in subinterval *I*_0_, the lowest point can be in any subinterval *I_n_*, depending on the nonlinear parameter *p*. If the orbit is in subinterval *I*_1_, then ascents and descents interchange with each other in each iteration. In the chaotic zone, this happens only for *p* ≤ *Q*_1_, while there is only one point with this behavior and that is the band-merging *p* = *Q*_1_. Since there is just one point in SCB that belongs in subinterval *I*_1_, then this must be *u*_1_. Hence, the condition that applies in the case of band merging (or, primary node *N*_0_) is:

(7a) $$u_{1}(p)=x_{\min}(p),\;\mathrm{or}\;p^{4}-4p^{3}+16=(p-2)\cdot (p^{3}-2p^{2}-4p-8)=0$$

where we find

(7b) $$p=Q_{1}=N_{0}=\frac{8}{3}{\left(3\sqrt{33}+19\right)}^{-\frac{1}{3}}+\frac{4}{3}+\frac{2}{3}{\left(3\sqrt{33}+19\right)}^{\frac{1}{3}}=3.678573510428329\ldots$$

The three peaks, shown in Figure 8a, represent the most frequent visits of the chaotic orbits for *p* = *Q*_1_ (primary node *N*_0_). Starting from the peak near the highest *x*-value, the orbits are mapped to the peak near the lowest *x*-value, and then, are mapped to the peak near the fixed point *u*_0_. The fact that there is only one ascent before reaching *u*_0_ indicates that the lowest *x*-value is actually the preimage *u*_1_, verifying Equation (7a).

Similarly, the four peaks in Figure 8b represent the most frequent visits of the chaotic orbits at primary node *N*_1_. Orbits from near the highest *x*-value are mapped to the peak near the lowest *x*-value, then mapped to one more peak before are mapped to the peak near the fixed point *u*_0_. The three sequential ascents indicate that the lowest *x*-value is actually the preimage *u*_2_. Hence, the condition that applies in the case of the primary node *N*_01_ is:

(8a) $$u_{2}(p)=x_{\min}(p),\;\mathrm{or}\;\frac{1}{2}\cdot (1-\sqrt{1-4/p^{2}})=\frac{1}{16}p^{2}(4-p)$$

where we find

(8b) $$p=N_{1}=3.927737001786751\ldots$$

In the case of the primary node *N_n_*, we have

(9a) $$p=N_{n}:\;u_{n+1}(p)=x_{\min}(p),\;{{f^{-1}}_{L}}^{\left(n+1\right)}(1-1/p;p)=f^{\left(2\right)}(\frac{1}{2};p)=\frac{1}{16}p^{2}(4-p)$$

Note that is equivalent $u_{n+1}=x_{\min}$ to ${f^{-1}}_{R}(u_{n+1})=x_{\max}$ and ${{f^{-1}}_{R}}^{\left(2\right)}(u_{n+1})=\frac{1}{2}$. Hence, we obtain

(9b) $$f^{\left(n+3\right)}(\frac{1}{2};p)=u_{0}\;\mathrm{or}\;{{f^{-1}}_{R}}^{\left(2\right)}{{f^{-1}}_{L}}^{\left(n+1\right)}(u_{0};p)=\frac{1}{2},\;u_{0}=1-1/p$$

Nonetheless, Equation (9a) is the one used for deriving the values of *N_n_*.

**Figure 10:**
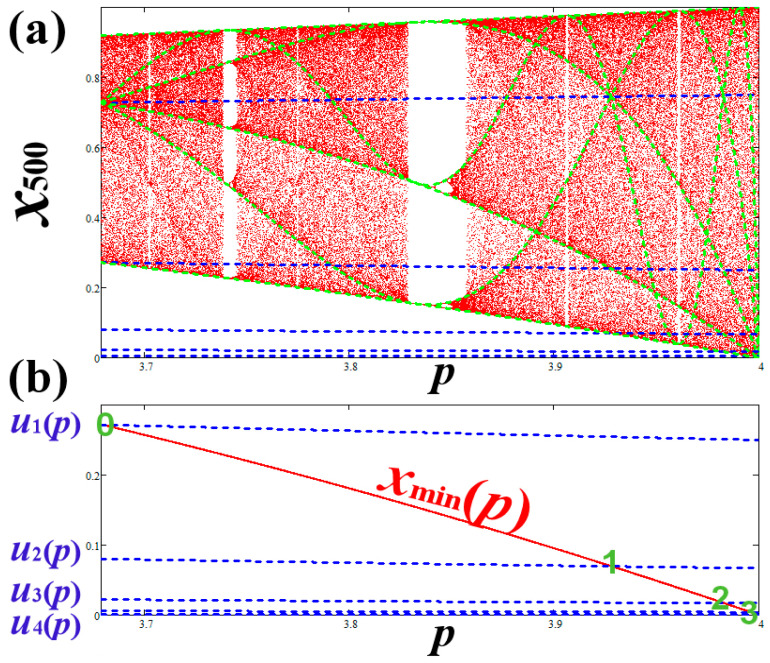

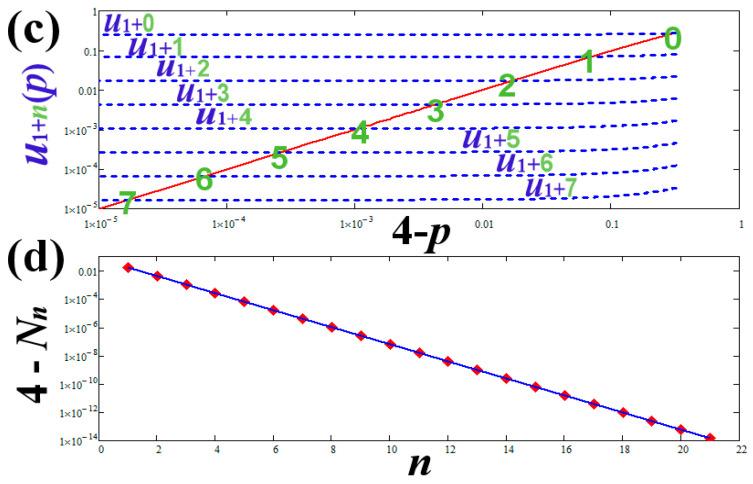
(**a**) Plot of SCB with critical curves (green) and preimages (blue). (**b**) The intersections of the preimages un with the lowest *x*-value give the *p*-value of the primary nodes. (**c**) The same as (**b**) but on log–log scale and the horizontal axis is 4 − *p*. (**d**) Plot of 4 − *N_n_* vs. the nodal order *n*; we observe that the primary nodes approach *p* = 4 with a geometric sequence (Equation (10)).

In Figure 10a, we plot the chaotic zone in SCB with the critical curves $X(p;n)=f^{\left(n+1\right)}\left(\frac{1}{2};p\right)$ (green) and preimages $u_{n}={{f^{-1}}_{L}}^{\left(n\right)}(u_{0})$ (blue), while in Figure 10b we show the intersections between $x_{\min}(p)=f^{\left(2\right)}(\frac{1}{2};p)$ and $u_{n}={{f^{-1}}_{L}}^{\left(n\right)}(u_{0})$, for *n* = 0, 1, 2, 3. The intersections give the *p*-values of the primary nodes, which are plotted on a logarithmic scale in Figure 10c. We observe that the ratio of the *p*-values between two sequential nodes is constant and equal to 4. This is also shown in Figure 10d, where we plot 4 − *N_n_* as a function of *n*. We find that

(10a) $$4-N_{n}=A\cdot 4^{-n}$$

where the constant is *A* ≈ 0.06853892. In [16], we have shown that for *p* = 4 the preimages are given by $u_{n+1}=\sin^{2}(\frac{\pi }{12}\cdot 2^{-n})\approx (\pi^{2}/144)\cdot 4^{-n}$ (cf., Equation (48c) in that paper), where $(\pi^{2}/144)\approx 0.06853892$; hence, it appears that the *p*-value of the primary nodes are connected with the preimages in the case of the fully-developed chaos (*p* = 4):

(10b) $$4-N_{n}\approx {u_{n+1}|}_{p=4}$$

## 5. Connection between WOMs and Nodes

The primary nodes, defined by *N*_0_ < … < *N_n_* < *N_n_*_+1_ < … < *N_∞_*, are not the only ones appear in the chaotic zone. There is another more complicated configuration of secondary nodes. These nodes appear always in pairs surrounding WOMs. All the secondary nodes surrounding a WOM have the same order; the latter is also equal to the WOM’s period.

**Figure 11:**
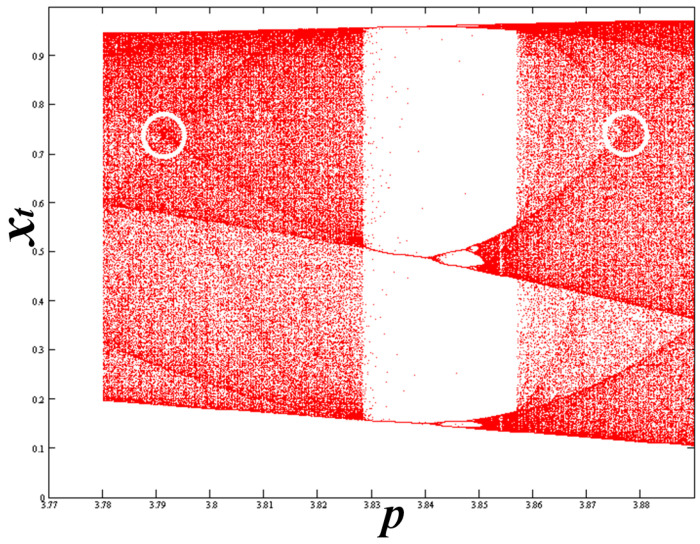
The pair of secondary nodes of order 3 surrounding the WOM of the same period in SCB.

The number of pairs of secondary nodes surrounding each WOM depends on the location with respect to the primary nodes. In the region between the primary nodes of order 0 and 1, called pyramid-1, the WOMs are surrounded by only one pair of secondary nodes; also, in the region between the primary nodes of order 1 and 2, called pyramid-2, the WOMs are surrounded by two pairs of secondary nodes, etc. For example, there is only one pair of secondary nodes surrounding the WOM of period three (Figure 11), but there are two pairs of secondary nodes surrounding the WOM of period four, etc. In general, the pyramid-*n* is defined by the region between the primary nodes of order *n* − 1 and *n*, the included WOMs are surrounded by *n* pairs of secondary nodes with order *n*, and the WOM of minimum period is the one with period *n* + 2, which is called main WOM of the pyramid.

Therefore, we have the following equalities:

*Order of secondary nodes* = *Period of surrounded WOM* (11a)

and

*Number of secondary nodal pairs* = *Order of next primary node* = *Period of main WOM* − 2 (11b)

**Figure 12:**
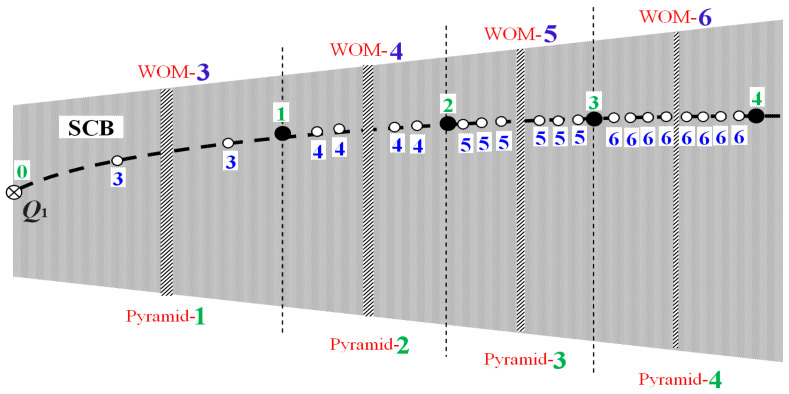
Sketch of the main chaotic band, SCB, of the chaotic zone. We show the location of the first four pyramids, their associated primary nodes, and the secondary nodes surrounding their main WOM. All the nodes, primary and secondary ones, are located at the orbit of period 1, *x*_∞_(*p*) = 1 − 1/*p* (dash line). The period of the main WOM (blue) is the same as the order of the surrounding secondary nodes. The number of the pairs of the surrounding secondary nodes is the same as the order of the next primary node (green).

The two secondary nodes of each pair are located one at each side of the surrounded WOM; for example, the main WOM in pyramid-3, that is, the WOM of period 5, is surrounded by 2 × 3 secondary nodes of order 5, where 3 of those are located at the left-hand side and 3 at the right-hand side of that WOM. Table A2 in Appendix A shows the secondary nodes up to the 6th order located within the main chaotic band, SCB. Figure 12 shows a sketch of SCB, where the primary and secondary nodes are shown.

**Figure 13:**
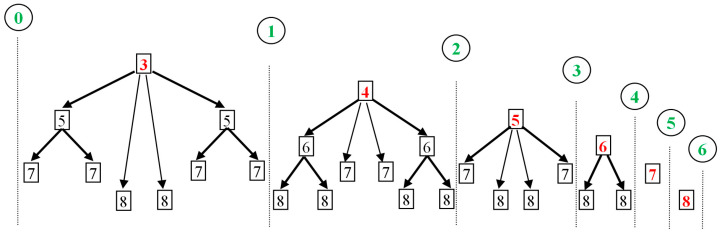
Sketch of the universal WOM-arrangement. Between any two consecutive primary nodes (green), WOMs are arranged in a pyramidal configuration: each WOM is surrounded by pairs of other WOMs with higher period, that is. The main WOMs (those with minimum period) are in the top of the pyramids (red).

In every CB, WOMs have a specific arrangement, universal for all the one-dimensional unimodal maps (due to a property called structural universality, [24,26,27,47]). Under each main WOM there is a pyramidal configuration of infinite number of WOMs: Every WOM is surrounded by infinite number of WOM pairs, whose period is larger than the parental WOM’s period, at least by two units. For example, the main WOM of period 3 is surrounded by pairs of WOMs of period 5, 7, 8, and so on, while period 6 does not exist and the same holds for any multiple of 3, i.e., the main WOM’s period. Similarly, the main WOM of period 4 is surrounded by pairs of WOMs of period 6, 7, and so on, while WOMs of period 8, or of any other multiple of 4, do not exist. Table A3 in Appendix A gives the location of WOMs (nonlinear parameter at their starting point), in the main chaotic band (SCB) and for period up to 0. The sketch in Figure 13 describes the arrangement of WOMs in CBs. It is interesting that the secondary nodes follow the same exact scheme as WOMs; the only difference is that nodes come in pairs surrounding each WOM.

## 6. Features of Nodes

The primary or secondary nodes can be the limit of one or more WOM sequences. Indeed, the locations (along the axis of the nonlinear parameter *p*) of the WOMs on the left-hand side of pyramid-*n* and of the WOMs on the right-hand side of pyramid-(*n* − 1) construct two sequences that both lead to the primary node *n*.

**Figure 14:**
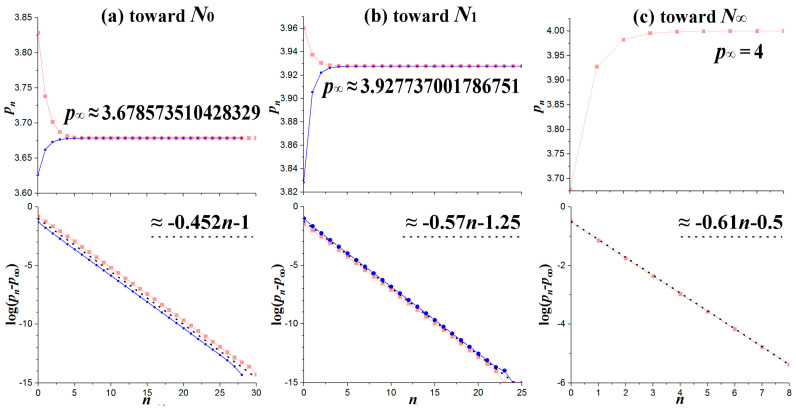
Convergence towards primary nodes: (**a**) *N*_0_; (**b**) *N*_1_; and (**c**) *N*_∞_ (upper panels); and their exponential rates (lower panels).

For example, the location of the primary node *N*_0_ (at the band-merging *Q*_1_) can be computed (aside from the mathematical formulation given in Section 4) by finding all the WOMs with odd periods starting from the WOM-3 along the left-hand side of the pyramid-1 (that is by decreasing the nonlinear parameter *p*). Alternatively, the same node can be computed by finding all the main WOMs of generation 1, i.e., along the chaotic bands CB(0) and CB(1), (that is by increasing the nonlinear parameter *p*). Both sequences are given in Table A4 in Appendix A, and plotted in Figure 14a (upper panel), where we observe that they both converge to the primary node *p*_∞_ → *N*_0_, while the exponential convergence of WOMs locations, ${\left\{p_{n}\right\}}_{1}^{\infty }$, toward the node is shown in the lower panel.

Similarly, the primary node *N*_1_ can be computed by finding the WOMs with even periods starting from the WOM-4 along the left-hand side of the pyramid-2 (that is by decreasing *p*) and the WOMS with odd periods starting from the WOM-3 along the right-hand side of the pyramid-1 (that is by increasing *p*). The two sequences are given in Table A5 in Appendix A, and plotted in Figure 14b (upper panel), where we observe that they both converge to the primary node *p*_∞_ → *N*_1_, while the exponential convergence of WOMs locations, ${\left\{p_{n}\right\}}_{0}^{\infty }$, toward the node is again shown in the lower panel. The latter is described by

(12) $$p_{n}≅p_{\infty }+A\cdot 10^{-\lambda \cdot n}$$

with log A ≈ −1 and λ ≈ 0.452 for *N*_0_, and log A ≈ −1.25 and λ ≈ 0.57 for *N*_1_.

We also examine how the primary nodes *N_n_* converge to the full-developed chaos at *p* = 4, which corresponds to the node *N*_∞_ (Table A1). The convergence is shown in Figure 14c (upper panel) with the exponential rate described by log A ≈ −0.5 and λ ≈ 0.61 for *N*_∞_ (lower panel).

The Feigenbaum constant *δ* is defined as the limit of the following sequence ${\left\{F_{n}\right\}}_{0}^{\infty }$:

(13) $$F_{n}\equiv \frac{p_{n}-p_{n-1}}{p_{n+1}-p_{n}},\;\delta =\lim_{n\to \infty }F_{n}$$

**Figure 15:**
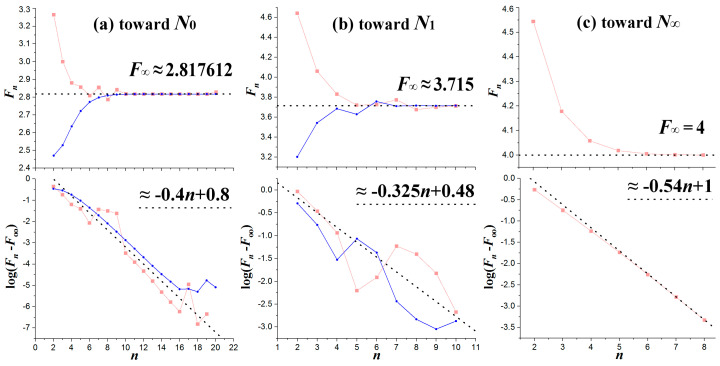
Convergence of Feigenbaum sequences ${\left\{F_{n}\right\}}_{0}^{\infty }$ toward their limit, i.e., the Feigenbaum constant $\delta =F_{\infty }$, for the sequences toward the primary nodes: (**a**) *N*_0_; (**b**) *N*_1_; and (**c**) *N*_∞_, shown in Figure 14 (using the tables in Appendix A). We observe the convergence toward the Feigenbaum constant (upper panels), and the corresponding exponential rates (lower panels).

The constant and its derivation was introduced by Feigenbaum [3,4,5,64] to describe the convergence of bifurcation points in the zone of order, but the same holds for the band-merging reverse cascade (e.g., [13]). In general, it can be applied to any convergence sequence in the chaotic zone. In Figure 15, we plot the computed Feigenbaum sequences ${\left\{F_{n}\right\}}_{0}^{\infty }$ and their limits, the Feigenbaum constant *δ*, which correspond to the convergence sequences plotted in Figure 14. The convergence to $\delta =F_{\infty }$ is shown in the upper panels of Figure 15, while the exponential rate of the convergence is shown in the lower panels.

We find that the Feigenbaum constants corresponding to the sequences converging toward the primary nodes *N*_0_ and *N*_1_ are *δ* ≈ 2.817612 and *δ* ≈ 3.715, respectively. It is worth mentioning that the same constant characterizes any of the two sequences, which approach the nodes from the left or from the right. Moreover, we find that the convergence of the sequence of primary constants toward *N*_∞_ is characterized by a peculiar Feigenbaum constant, that is, exactly *δ* = 4.

Finally, we examine the entropy near the nodes. Given the distribution density at *p*, P(x;p), the entropy in the continuous description is given by the standard Shannon’s form [65]:

(14) $$S(p)=-\int_{0}^{1}P(x)\cdot \ln[P(x)\cdot \sigma_{x}]dx,\;\mathrm{where}\;\int_{0}^{1}P(x)dx=1$$

where $\sigma_{x}$ is the smallest scale that characterizes the physical quantity *x* (e.g., see: [66,67]).

The discretization of this interval is given by setting

(15) $$P_{i}=P(x_{i})\cdot \sigma_{x},x_{i}=i\cdot \sigma_{x}$$

hence, the entropy is given by

(16) $$S(p)=-\sum_{i=0}^{\left[1/\sigma_{x}\right]}P_{i}\ln P_{i}$$

where $\left[1/\sigma_{x}\right]$ denotes the closer integer to $1/\sigma_{x}$.

**Figure 16:**
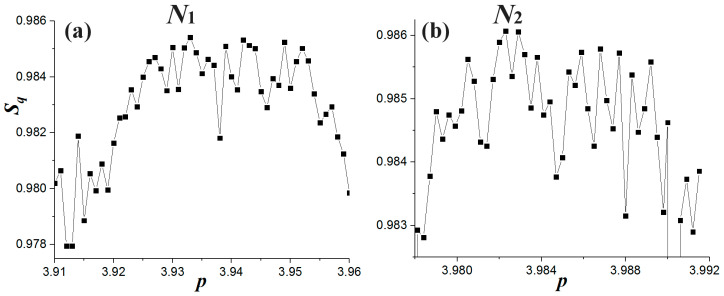
Entropy *S_q_* computed and plotted for entropic index *q* = 2, [1/*σ_x_*] = 100, and nonlinear parameter *p* values taken near the primary nodes *N*_1_ and *N*_2_.

We also use another entropic form, generalized according to the Tsallis mono-parametrical formalism [68,69],

(17) $$S_{q}(p)=\frac{1}{q-1}\cdot \left(1-\sum_{i=0}^{\left[1/\sigma_{x}\right]}{P_{i}}^{q}\right)$$

where recovers the Shannon’s entropy in Equation (16) for *q* → 1. We find the entropy is ~1 at the primary nodes for *q* ~ 1.985. We chose this specific *q*-index for computing the entropy, as for smaller *q*-indices, the entropy increases abruptly, while for larger *q*-indices it tends to zero. We find that the entropy increases as the nonlinear parameter approaches the nodes. Figure 16 plots the entropy calculated for *q* = 2 and [1/*σ_x_*] = 100, where we observe that it increases when *p* is taken closer to the primary nodes *N*_1_ and *N*_2_.

## 7. Conclusions

The paper presented the arrangement and features of nodes in the chaotic zone of 1D unimodal and mono-parametrical discrete maps *f*(*x*), using as guiding example the logistic map. The nodes are high density and intersection points of the critical curves in the chaotic zone.

First, we examined the arrangement of critical curves in the chaotic zone, using the density of the variable *x*, and a simple empirical formula that describes these curves.

Second, we examined the identification and arrangement of nodes in the chaotic zone. The peaks of density profiles, taken across the nonlinear parameter axis, reveal the position of critical curves; as the nonlinear parameter approaches to a node, these peaks converge to each other, forming only a certain number of peaks that defines the order of the node. We found two types of nodes. (i) The primary nodes are defined by their unique sequential arrangement in the chaotic band: There is only one primary node of order *n* + 1 beyond the primary node of order *n* (along the nonlinear parameter axis). This arrangement separates chaotic bands in regions called pyramids. These are specific configurations of WOMs. (ii) The secondary nodes appear in pairs surrounding WOMs, where the order of nodes equals the period of the surrounded WOMs. The number of pairs is a characteristic of the pyramid.

Third, we examined the connection between WOMs and nodes. There is a universal arrangement of WOMs in chaotic bands, which can be divided into pyramidal configurations, separated by the primary nodes.

Finally, we examined several features of nodes, such as the convergence and entropy. We computed the characteristic convergence rates of the sequences of WOMs that converge into primary nodes, as well as the sequence of primary nodes that converge to the point of full-developed chaos. We computed the Feigenbaum constants related to these convergences, showing that each node has its characteristic Feigenbaum constant. The entropy analysis revealed that near the nodes the entropy has a local maximum value.

The following related science questions may be examined in future analyses: How do the arrangement and features of nodes vary for S-unimodal maps with a local maximum of differential order other than 2 (e.g., [13,31,58])? What is the mathematical formulation that describes the secondary nodes? Are there types of nodes other than the primary and secondary ones? Are there the same features of nodes into the 1D Poincare section return maps (e.g., [70,71])? How the nodes and their properties appear in higher dimensions (e.g., [72,73])?

## Appendix A. Tables

**Table A1:** Order and nonlinear parameter of primary nodes.

Order	*p*
0 *	3.678573510428329
1	3.927737001786751
2	3.982570733172925
3	3.995693633605350
4	3.998927382603362
5	3.999732146052877
6	3.999933058608207
7	3.999983266242305
8	3.999995816673156

* Note: It corresponds to the band-merging of SCB at *Q*_1_.

**Table A2:** Order and nonlinear parameter of secondary nodes *.

3 Order	4 Order	5 Order	6 Order
3.791097, 3.876540	3.946550, 3.972211	3.727254, 3.752684	3.933204, 3.941763
-	-	3.894663, 3.915998	3.9751854, 3.9801912
-	-	3.9867746, 3.9929707	3.9967110, 3.9983206

* Note: We provide the location of the first and last secondary nodes surrounding each WOM.

**Table A3:** Nonlinear parameter of WOMs (starting point) in SCB for period ≤9.

3	4	7	8	9	(Cont.)
3.82843	3.960102	3.70164	3.80074	3.6872	3.975919
**5**	**6**	3.77413	3.87053	3.7171	3.979543
3.73817	3.93752	3.88602	3.89946	3.76124	3.983140
3.90557	3.977765	3.922186	3.91205	3.78577	3.986274
3.99026	3.9975826	3.951027	3.93047	3.87941	3.989188
		3.968974	3.94421	3.89225	3.991324
		3.984746	3.97372	3.917792	3.993577
		3.9945375	3.981408	3.926277	3.995417
		3.9993970	3.987746	3.9346999	3.996945
			3.992519	3.94037	3.998148
			3.9962195	3.947735	3.999058
			3.9986417	3.954483	3.999661
			3.9998495	3.966193	3.9999624
				3.971413	

**Table A4:** Convergence to the primary node of order 0.

Left	*p*	Right	*p*
2 × 3	3.62656	3	3.82842712
2 × 4	3.66211	5	3.73817237
2 × 5	3.67300	7	3.70164076
2 × 6	3.67663	9	3.68719687333
2 × 7	3.67789	11	3.68171601937
2 × 8	3.678331	13	3.67970245777
2 × 9	3.678488	15	3.67897629733
2 × 10	3.678543	17	3.678716777082
2 × 11	3.67856274217796	19	3.6786244025542
2 × 12	3.678569688994185	21	3.67859157901113
2 × 13	3.678572154206041	23	3.67857992406206
2 × 14	3.678573029096640	25	3.678575786820828
2 × 15	3.6785733395993465	27	3.6785743183617855
2 × 16	3.6785734497993825	29	3.6785737971749995
2 × 17	3.6785734889104695	31	3.6785736121981435
2 × 18	3.678573502791405	33	3.6785735465475785
2 × 19	3.678573507717898	35	3.6785735232474435
2 × 20	3.6785735094663632	37	3.678573514977968
2 × 21	3.678573510086913	39	3.678573512043041
2 × 22	3.678573510307152	41	3.678573511001403
2 × 23	3.678573510385	43	3.6785735106317145
2 × 24	3.678573510413	45	3.678573510500508
2 × 25	3.678573510423	47	3.678573510453941
2 × 26	3.6785735104264	49	3.6785735104374145
2 × 27	3.67857351042764	51	3.678573510431549
2 × 28	3.678573510428080	53	3.678573510429467
2 × 29	3.678573510428237	55	3.678573510428729
2 × 30	3.678573510428292	57	3.678573510428467
2 × 31	3.678573510428312	59	3.678573510428373
		61	3.678573510428340
		63	3.678573510428329

Note: The sequence of main WOMs in CB(0) and CB(1) (CBs of generation *n* = 1) converges from the left to the primary node of order 0 (*N*_0_, that is, the band-merging *Q*_1_). The sequence of WOMs on the left side of the pyramid-1 in SCB (CB of generation *n* = 0) converges from the right to the primary node of order 0 (*N*_0_).

**Table A5:** Convergence to the primary node of order 1.

Left WOMs	*p*	Right WOMs	*p*
3	3.82843	4	3.96010
5	3.90557	6	3.93752
7	3.922186	8	3.93047
9	3.926277	10	3.92848
11	3.927345	12	3.92794
13	3.927632	14	3.9277912
15	3.927709	16	3.9277516
17	3.9277294	18	3.92774093
19	3.92773495	20	3.927738059
21	3.92773645	22	3.927737286
23	3.927736854	24	3.927737078
25	3.927736962	26	3.92773702239
27	3.927736991	28	3.92773700733
29	3.9277369989	30	3.92773700328
31	3.92773700101	32	3.9277370021882
33	3.92773700158	34	3.9277370018948
35	3.927737001731	36	3.9277370018158
37	3.927737001772	38	3.9277370017946
39	3.92773700178267	40	3.927737001788857
41	3.92773700178565	42	3.927737001787318
43	3.92773700178646	44	3.927737001786904
45	3.927737001786673	46	3.927737001786793
47	3.927737001786730	48	3.927737001786763
49	3.927737001786746	50	3.927737001786755
51	3.927737001786750	52	3.927737001786753
53	3.927737001786751	54	3.927737001786751

Note: The sequence of main WOMs in CB(0) and CB(1) (CBs of generation *n* = 1) converges from the left to the primary node of order 1 (*N*_1_). The sequence of WOMs on the left side of the pyramid-1 in SCB (CB of generation *n* = 0) converges from the right to the primary node *N*_1_.
